# Status of the anaplastic lymphoma kinase (ALK) gene in inflammatory breast carcinoma

**DOI:** 10.1186/2193-1801-2-409

**Published:** 2013-08-28

**Authors:** Savitri Krishnamurthy, Wendy Woodward, Wei Yang, James M Reuben, James Tepperberg, Dai Ogura, Shin-ichiro Niwa, Lei Huo, Yun Gong, Randa El-Zein, Ana M Gonzalez-Angulo, Mariana Chavez-MacGregor, Ricardo Alvarez, Anthony Lucci, Vicente Valero, Naoto T Ueno

**Affiliations:** Department of Pathology, The University of Texas MD Anderson Cancer Center, 1515 Holcombe Blvd., Unit 53, Houston, TX 77030 USA; Department of Radiation Oncology, The University of Texas MD Anderson Cancer Center, Houston, TX USA; Department of Breast imaging, The University of Texas MD Anderson Cancer Center, Houston, TX USA; Department of Hematopathology, The University of Texas MD Anderson Cancer Center, Houston, TX USA; Cytogenetics Laboratory, LabCorp, Durham, NC USA; Department of Genomics, Link Genomics, Tokyo, Japan; Department of Epidemiology, The University of Texas MD Anderson Cancer Center, Houston, TX USA; Department of Breast Medical Oncology, The Morgan Welch Inflammatory Breast Cancer Research program and clinic, The University of Texas MD Anderson Cancer Center, Houston, TX USA; Department of Breast Surgical Oncology, The Morgan Welch Inflammatory Breast Cancer Research program and Clinic, The University of Texas MD Anderson Cancer Center, Houston, TX USA

## Abstract

**Background:**

Although preliminary reports suggest that *ALK* gene amplification may occur in inflammatory breast cancer (IBC), data are limited. We performed a comprehensive investigation of the status of *ALK* gene in IBC.

**Methods:**

We used core biopsy (CB) samples from 30 IBC patients for immunohistochemistry (IHC), 25 of these samples for fluorescence in situ hybridization (FISH) of *ALK* gene rearrangement, 8 for chromosome 2 aneusomy, and 20 microdissected frozen CBs for array comparative genomic hybridization (CGH) and mRNA analysis.

**Results:**

All 30 samples were negative for ALK protein expression by IHC. FISH analysis showed no *EML4-ALK* gene rearrangement in any samples, although 16 of the 25 samples (64%) contained 3 to 4 extra copies of the *ALK* gene, and chromosome 2 aneusomy was found in 7 of 8 samples that had extra copies of the *ALK* gene. Only 3 of the 20 samples showed evidence of mild *ALK* gene amplification by array CGH. mRNA analysis revealed that mRNA expression of *ALK* was not significantly higher in these samples compared with samples that showed no evidence of *ALK* gene amplification in CGH analysis, nor was mRNA expression of *ALK* significantly different in tumor compared with 5 normal breast samples (*P* > 0.05, *t* test).

**Conclusion:**

Our comprehensive evaluation suggests that *ALK* gene rearrangement did not occur in the IBC patients studied. The significance of our finding of mildly increased copy numbers of the *ALK* gene resulting from chromosome 2 aneusomy rather than mild amplification of the *ALK* gene requires further investigation.

## Introduction

The anaplastic lymphoma kinase (*ALK*) gene belongs to the insulin receptor superfamily and encodes a receptor tyrosine kinase that is normally expressed in select neuronal cell types (Pulford et al. 1997 Morris et al. ;^1997^; Iwahara et al. 1997). Aberrant *ALK* activity resulting from point mutations, amplifications, chromosomal translocations, or other types of rearrangements has been implicated in the pathogenesis of selected human cancers. *ALK* was first identified as a fusion partner of nucleophosmin in anaplastic large-cell lymphoma resulting from t(2,5)(p23;q35) chromosomal translocation (Morris et al. 1994; Shiota et al. 1994). Chromosomal translocations linking *ALK* to other fusion partners in anaplastic large-cell lymphoma, inflammatory myofibroblastic tumors, and neuroblastomas have been established subsequently (Pulford et al. 2004; Lawrence et al. 2000; George et al. 2008; Chen et al. 2008; Janoueix-Lerosey et al. 2008; Mossé et al. 2008).

Recently, a novel gene fusion involving *ALK* and echinoderm microtubule-associated protein-like 4 (*EML4*) was discovered in non-small cell lung carcinoma (NSCLC) (Soda et al. 2007; Mano 2008). This gene fusion was reported to result from a small inversion within the short arm of chromosome 2 involving an almost identical portion of *ALK* exons (20–29) representing the intracellular domain of the molecule and various portions of the *EML4* exons (Soda et al. 2007; Wong et al. 2009; Takeuchi et al. 2008; Choi et al. 2008; Koivunen et al. 2008; Takeuchi et al. 2009). The presence of intact ALK kinase domain in a fusion protein resulting from *ALK* rearrangement results in transformation as well as oncogenic activity in the cells. (Soda et al. 2007; Choi et al. 2008; Soda et al. 2008).

Treatment with ALK inhibitors in vitro has been reported to lead to cell cycle arrest and apoptosis in anaplastic large-cell lymphoma, NSCLC, and neuroblastoma cells with any of the noted *ALK* rearrangements (Wan et al. 2006; Christensen et al. 2007; Galkin et al. 2007; McDermott et al. 2008). Multiple small-molecule ALK inhibitors have been developed to antagonize cells with the EML4-ALK fusion.

Although *EML4-ALK* gene rearrangement has been studied in solid tumors such as NSCLC, few studies have investigated their role in breast cancers. A recent report described a possible role for *ALK* amplification in inflammatory breast cancer (IBC) (Robertson et al. 2011). Recent research has focused on understanding the genomic makeup of IBC to account for its distinct clinical presentation and biology compared with other types of breast cancer. An intense effort is also underway to unearth possible targets for currently available therapeutic agents so that patient outcomes can be improved with this very aggressive variant of breast cancer. However, the role of *ALK* gene and the implications of *ALK* amplification in IBC for available targeted treatments using small-molecule ALK inhibitors remain unclear.

To address all these knowledge gaps, we conducted a comprehensive investigation of *ALK* gene in tumor samples from IBC patients using immunohistochemistry (IHC) to evaluate ALK protein expression, fluorescence in situ hybridization (FISH) for EML4-ALK rearrangement, and array comparative genomic hybridization (CGH) and transcriptional profiling to evaluate copy number and mRNA levels of the *ALK* gene.

## Materials and methods

### Tumor samples

We used ultrasound-guided core needle biopsy (CNB) tumor samples from 30 IBC patients under an Institutional Review Board (The University of Texas MD Anderson Cancer Center) approved protocol. The primary invasive ductal carcinoma was categorized as Nottingham histologic grade 2 in 12 of the 30 patients and as grade 3 in the remaining 18 patients. Tumor samples from 12 patients were positive for estrogen (ER) or progesterone receptors (PR) but negative for human epidermal growth factor receptor 2 (HER2), samples from 13 patients were negative for ER and PR but positive for HER2, and samples from 5 patients were negative for all 3 markers.

The CNB’s were fixed in formalin, routinely processed, embedded in paraffin wax, and cut into 5-μm unstained tissue for IHC staining and FISH. They were embedded in optimal cutting medium (OCT), cut into 5-μm sections and stained with hematoxylin and eosin to confirm the presence of invasive carcinoma with sufficient cellularity (at least 75%) to be used for CGH and mRNA analysis.

### IHC staining for ALK

Immunostaining was performed in a Bond max automatic immunostainer (Leica). Heat-induced epitope retrieval was performed on deparaffinized tissue sections using Tris-EDTA buffer (pH 6.0). Primary monoclonal ALK antibody (Dako™ Cytomation, Carpentaria, CA) at 1:25 dilution was applied on the tissue sections. Endogenous peroxide was blocked using 3.0% hydrogen peroxide and polymer horseradish peroxidase anti-mouse/anti-rabbit IgG was applied on the slides. Diaminobenzidine was added as the chromogen for signal recognition. Positive staining was recognized as granular brown cytoplasmic positivity in the tumor cells.

### FISH for ALK gene rearrangement

To detect *ALK* gene rearrangement, we used the EML4-ALK breakapart probe (Abbot™Laboratories, Des Plaines, IL) in 25 of the samples. Unstained tissue sections were baked for 24 hours at 56°C in a ThermoBrite temperature controlled slide processing system (Abbott Molecular Inc., Des Plaines, IL) and immersed in Hemo-De solution. The slides were immersed in Vysis™ pretreatment solution (1 M sodium thiocyanate) at 80°C for 10 minutes, and in protease solution (previously warmed to 37°C) for 10 minutes, washed with purified water, air-dried, and dehydrated in ascending grades of alcohol.

Ten microliters of the ALK probe mixture was applied on the air-dried slides. Denaturation and hybridization of the tissue sections was performed using the Thermobrite system (Abbott Molecular Inc. Des Plaines, IL), which was programmed to 75°C for 5 minutes for the denaturation process and 37°C for 16 hours for the hybridization of the probes. The slides were then washed with 0.4X saline-sodium citrate (SSC) solution at 70°C for 2 minutes and 2X SSC at room temperature for 3–5 minutes. Lastly, 10 μL of DAPI was applied on the slides; the slides were examined using a fluorescent microscope.

FISH signals were counted in 50 tumor cells. The breast tumor was considered positive for EML4-ALK rearrangement when at least 1 set of orange and green signals were 2 or more signals apart, a single orange signal was observed without a corresponding green signal, or fused or broken apart signals were observed in 15% of the tumor cells. The breast tumor was considered negative for EML4-ALK rearrangement when the orange and green signals were adjacent or fused (appeared yellow), orange and green signals were less than 2 signals apart, or a single green signal was observed without a corresponding orange signal.

### FISH for CEP2

To detect aneusomy in chromosome 2, we performed FISH using the CEP2 probe (Abbott Molecular™) in 8 samples with increased EML4-ALK signals. Five micron tissue sections were de-paraffinized and pretreated for 80 minutes and 30 minutes (pretreatment and protease, respectively) using a VP 2000 (Abbott Molecular™, Des Plaines, IL) instrumentation.

Slides were then pre-aged in 2X SSC/0.05% IGEPAL, pH 7.0, for 5 minutes and dehydrated in 70%, 80%, and 100% cold ethanol for 1 minute each and then dried at 37°C for 5 minutes. 10 ul CEP 2 was placed on the target area and co-denatured at 72.0°C for 6.5 minutes and hybridized for 8 hours at 37°C, using a Thermobrite instrument. The slides were subsequently washed in 2XSSC/0.05% IGEPAL for 2 minutes at 74°C followed by 2XSSC/0.05% IGEPAL for 1 minute at room temperature. 18 ul of DAPI/AF was applied, coverslip placed, and analyzed using a Nikon Eclipse fluorescence microscope.

### Genomic DNA and RNA sample preparation

Twenty frozen tissue blocks were cut into 16-μm sections and manually dissected to obtain invasive tumor cells which was used for genomic DNA and RNA extraction. In addition, 40–60 frozen sections of benign breast tissue were used as controls for CGH and transcriptional profiling.

Genomic DNA was extracted from cells using the Quick Gene SP-kit (Fujifilm™, Tokyo, Japan), and quality was characterized using agarose gel electrophoresis. Human female genomic DNA (Novagen™, Billerica, MA) was used for normal reference DNA. Total RNA was extracted from the cells with RNeasy micro kit (Qiagen™, Germantown, MD), and quality was characterized using BioAnalyzer (Agilent Technologies™, Santa Clara, CA).

### Preparation of the whole-genome DNA microarray

We created a whole-genome DNA microarray with a tiling resolution that included complete coverage of the human genome, using 12,310 individually amplified bacterial artificial chromosome (BAC) clones. All BAC clones were cultured in a single colony and validated with polymerase chain reaction (PCR) amplification using clone-specific primers. BstYI was restricted in the extracted BAC DNA, and DNA was amplified using ligation-mediated PCR and printed on glass slides using an inkjet-type spotter.

### Array CGH analysis of genome copy number

Alu I and Rsa I-restricted genomic DNA was labeled by random priming with Alexa555-dCTP (test DNA) and Alexa647-dCTP (reference DNA) using the BioPrime Plus ArrayCGH Indirect Genomic Labeling System (Invitrogen, Macro Island, FL). Labeled test and reference DNA was precipitated with ethanol in the presence of Cot-1 DNA, redissolved in a hybridization mix, and denatured at 70°C for 10 minutes. After being incubated at 42°C for 5 minutes, the mixture was applied to the whole-genome DNA microarray (LinkGenomics, Tokyo, Japan) and covered with a MAUI mixer hybridization chamber (Biomicro Systems). After being incubated at 42°C for 48 hours, the slides were washed with 2X SSC/0.1% sodium dodecyl sulphate (SDS) buffer and 0.1X SSC/0.1% SDS buffer several times. The slides were then rinsed with 0.01× SSC buffer and air-dried, scanned with a Microarray Scanner (Agilent Technologies, Santa Clara, CA), and analyzed using Gene-Pix Pro 4.0 imaging software (Axon Instruments, Inverurie, Scotland). We performed normalization using global-normalization methods and calculated the fold changes of gene amplification compared with the normal tissue, focusing on 2 BAC clones (RP11-62B19 and RP11-701P18) covering the regions containing the *ALK* gene (2p23). A G/R ratio ≥1.2 indicated a gain, and a G/R ratio ≤0.8 indicated a loss.

### Microarray analysis of mRNA expression

Microarray analysis was performed using 20 tumor samples (the same samples used for CGH analysis) and 5 normal breast tissue samples. RNA samples were labeled using the Low Input Quick Amp Labeling Kit (Agilent Technologies, Santa Clara, CA). Labeled cRNA was hybridized to an oligonucleotide microarray (Whole Human Genome 4 × 44 K; Agilent Technologies) at 60°C for 17 hours. The slides were washed with the Gene Expression Wash Buffer Kit (Agilent Technologies, Santa Clara, CA), dried, then scanned with an Agilent Microarray Scanner and analyzed using the Feature Extraction software version 9.5.1 (Agilent Technologies, Santa Clara, CA). Normalization was performed using global-normalization methods. The mRNA expression was compared between tumor samples and normal breast tissue samples using student *t* test.

## Results

### IHC staining for ALK

**Figure 1:**
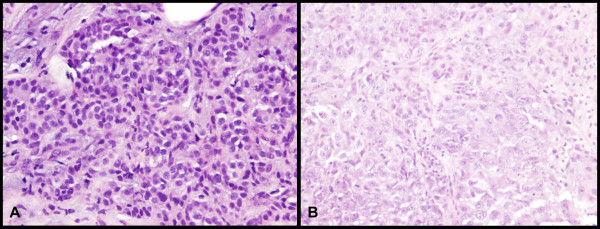
**Hematoxylin and eosin staining (magnification x200; A) and ALK immunohistochemical staining (magnification x200; B) in a representative sample of tumor tissue from a patient with inflammatory breast carcinoma.** Note that the tumor cells are entirely negative for ALK protein overexpression **(B)**.

All 30 primary tumor samples were negative for ALK protein overexpression. Figure 1 illustrates a representative case showing absence of ALK protein overexpression.

### FISH for ALK gene rearrangement and CEP2

None of the 25 samples showed any evidence of separation of EML4 (green signals) and ALK (orange signals) indicating the absence of *EML4-ALK* gene rearrangement. However, in 16 (64%) of the 25 samples, we found 3 to 4 extra copies of adjacently placed EML4 and ALK signals in 2-50% of the tumor cells. We evaluated 8 of these samples for aneusomy of chromosome 2 using the CEP2 probe.

**Figure 2:**
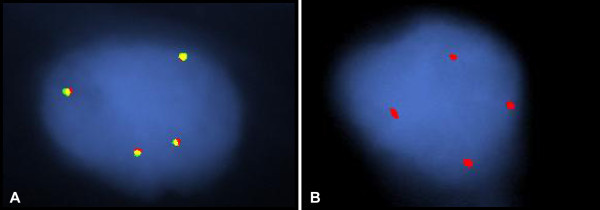
**Fluorescence in situ hybridization results in a representative sample of tumor tissue from a patient with inflammatory breast carcinoma.** Analysis using the Vysis ALK Break Apart probe **(A)** showed no evidence of fusion of the signals, but 2 extra copies of the *ALK* gene are present. Extra copies of CEP2 were also present **(B)**, indicating aneusomy of the tumor cells for chromosome 2.

The CEP2 analysis revealed evidence of aneusomy of chromosome 2 in 7 (88%) of the 8 samples examined, with 3–4 signals observed in 4-57% of the tumor cells. Figure 2 illustrates a representative sample showing a few extra copies of *ALK* (Figure 2A) associated with evidence of chromosome 2 aneusomy (Figure 2B).

### CGH analysis of genome copy number

Results of the CGH analysis using 2 BACs in the regions containing the ALK gene are shown in Table 1. In 17 (85%) of the 20 samples examined, we did not observe any gains of either of the 2 BAC clones (G/R ratio < 1.2), indicating that no extra copies of the *ALK* gene were present. A gain of only 1 BAC clone was observed in 2 samples and a gain of both BAC clones was observed in 1 sample. However, because the G/R ratios were low (<1.3), this finding suggests low levels of *ALK* gene amplification. In any case, *ALK* gene amplification was not present in most of the samples that we examined.

**Table 1:** **Results of array comparative genomic hybridization analysis of 20 tumor samples from patients with inflammatory breast carcinoma**

Sample no.	G/R ratio	
	RP11-62B19 BAC clone	RP11-701P18 BAC clone
1	1.11	1.1
2	1.03	1.03
3	0.94	0.93
4	0.99	0.98
5	1.15	1.25
6	1.01	0.96
7	0.94	0.93
8	1	0.99
9	0.83	0.86
10	0.84	0.89
11	1.05	1.06
12	1.1	1.11
13	1.17	1.19
14	1.08	1.08
15	1.17	1.14
16	1.03	1.04
17	1.27	1.29
18	0.85	0.85
19	1.11	1.14
20	1.24	1.18

For each bacterial artificial chromosome (BAC) clone, G/R ratios higher than 1.2 (indicating BAC clone gain and possible *ALK* gene amplification) are shown in boldface type.

### Microarray analysis of mRNA expression

**Figure 3:**
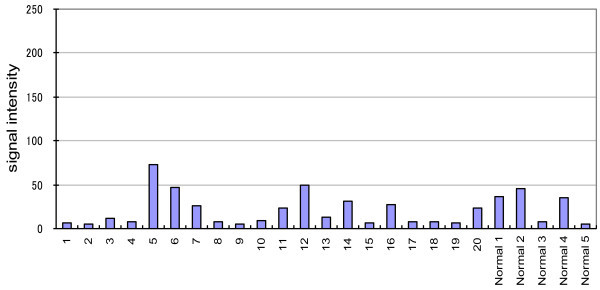
**Results of transcriptional profiling showing mRNA expression levels of the** ***ALK*** **gene in 20 tumor samples from patients with inflammatory breast carcinoma and 5 normal breast tissue samples.**

Results of transcriptional profiling are depicted in Figure 3. mRNA expression of the *ALK* gene was not significantly higher in the 20 tumor samples in comparison to the 5 normal breast tissue samples (p > 0.05, *t* test). The mRNA expression of *ALK* in the RNA obtained from the 3 samples that showed possible *ALK* gene amplification in the CGH analysis was not significantly higher than in the RNA from the 17 samples that did not show evidence of *ALK* gene amplification in the CGH analysis (p > 0.05, *t*-test).

## Discussion

Our comprehensive evaluation of the status of ALK gene did not reveal any evidence of *ALK* gene rearrangement, mRNA overexpression or increased protein levels in IBC. FISH analysis using dual color breakapart probes did not show any evidence of EML4-ALK gene rearrangements. A common occurrence in the FISH analysis of IBC tumors was however the presence of a mild increase in copy numbers of the EML4-ALK signals in a fraction of the tumor cells. The presence of increased copy numbers of the ALK gene in IBC can be due to either ALK gene amplification or aneusomy for chromosome 2, the parent chromosome on which the ALK gene resides. By extending the FISH analysis for EML4-ALK gene rearrangement with FISH for chromosome centromere 2 (CEP 2) we could further stratify patients in whom the increased copy numbers of the ALK gene was a result of chromosome 2 aneusomy as against those with true amplification of the ALK gene. Although the evaluation of the status of chromosome 2 was performed in only a small proportion of the IBC tumors, the finding of aneusomy for chromosome 2 in majority of the tested IBC patients (7/8) suggests that the finding of mildly increased copy numbers of the ALK gene by FISH resulted mostly from mildly increased copies of chromosome 2 and not due to ALK gene amplification. In rare patients with mildly increased copy numbers of the EML4-ALK with diploid status of chromosome 2 it is possible that there may be low levels of ALK gene amplification. It is to be noted that although we found mild increase in the copy numbers of the ALK gene in 64% of the IBC patients by FISH analysis, alterations of copy numbers of the ALK gene was detected in only 3 out of the 20 patients by CGH analysis. The reason for such discordance is most likely due to variable sensitivities of the DNA probes utilized in the EML4-ALK breakapart probes used in FISH and the 2 BAC clones used in CGH analysis. The absence of elevated mRNA levels in any of the patients by transcriptional profiling as well as the absence of ALK protein overexpression by IHC suggests that the functional significance of low-level amplification of the ALK gene that can occur in occasional patients with IBC without chromosome 2 aneusomy is perhaps limited. It is to be acknowledged that the ALK antibody used in the study may not be highly sensitive so as to detect low levels of ALK protein overexpression. However, the absence of elevated ALK mRNA levels in any of the IBC patients provides support that the low level of gene amplification in occasional IBC patients may not be of clinical significance. Nevertheless the possibility of triggering of the downstream signaling pathways in the tumor cells as a result of the mild increase in copy numbers of the ALK gene with or without underlying chromosome 2 aneusomy needs further investigation.

Very few studies have investigated the status of the *ALK* gene in breast cancer. Grob et al. studied driver mutations affecting the RAS/MAP kinase pathway in 65 samples of triple-negative breast cancers (Grob et al. 2012). They did not find any case of *ALK* gene rearrangements. Similarly, Fukuyoshi et al. studied EML4-ALK fusion transcripts using reverse transcriptase PCR and they did not find any EML4-ALK fusion transcripts (Fukuyoshi et al. 2008). The genomic types of the breast carcinoma that were investigated were not reported in this study.

To the best of our knowledge, only 1 study has reported EML4-ALK fusion in breast carcinoma. Lin et al. used profiling of cancer genomes on an exon array to develop a novel computational method for searching gene rearrangements in solid tumors (Lin et al. 2009). They detected EML4-ALK fusion in 5 of 209 breast carcinomas (2.4%). However, the genomic types of the breast cancers that demonstrated EML4-ALK fusion were not reported, nor is it known whether any of the 5 tumors that had the EML4-ALK fusion were associated with clinicopathologic features characteristic of IBC.

An abstract by Robertson et al. is to the best of our knowledge, the only report investigating the status of the *ALK* gene in IBC (Robertson et al. 2011). During a proteomic pathway analysis, the ALK pathway was found to be activated in 3 different IBC cell lines, and later genomic experiments showed that the *ALK* gene was amplified in IBC cell lines and in 9 of the 12 tumor samples analyzed. Activating mutations were not observed, but ALK amplification was found to be a prevalent finding in proteomic-genomic evaluation. The authors also reported that crizotinib, a small-molecule inhibitor of ALK, arrested growth of IBC cells in vitro and activated the cell death pathway. Moreover, xenograft IBC tumors were resistant to paclitaxel but sensitive to low doses of crizotinib, which resulted in tumor shrinkage similar to that reported in ALK-driven NSCLC models. *ALK* gene amplification was found to be a common feature of IBC, and the authors suggested that small-molecule ALK inhibitors could be effective in mouse xenograft IBC models. In a subsequent commentary related to the abstract published by Robertson et al., Tuma suggested that validation with a clinical trial was needed (Tuma 2012). However, our findings showing that the extra copies of the ALK gene observed in 64% of the IBC patients were largely associated with aneusomy of chromosome 2 led us to a different conclusion than that of Robertson et al. The results of our comprehensive evaluation of the status of ALK gene does not support the presence of EML4-ALK gene rearrangements in IBC. Our finding of mildly increased copy levels of the ALK gene in a majority of IBC patients resulting as a consequence of chromosome 2 aneusomy and not due to amplification of the ALK gene needs further validation in a larger cohort of IBC patients. The possible activation of downstream signaling pathways resulting from the presence of extra copies of the ALK gene in IBC warrants further exploration.
